# Self-learning of point-of-care cardiac ultrasound – Can medical students teach themselves?

**DOI:** 10.1371/journal.pone.0204087

**Published:** 2018-09-27

**Authors:** Lior Fuchs, David Gilad, Yuval Mizrakli, Re’em Sadeh, Ori Galante, Sergio Kobal

**Affiliations:** 1 Joyce and Irwing Goldman Medical School, Ben-Gurion University, Beer-Sheva, Israel; 2 Medical Intensive Care Unit, Soroka University Medical Center and The Faculty of Health Sciences, Ben-Gurion University of the Negev, Beer-Sheva, Israel; 3 Clinical Research Center, Soroka University Medical Center and The Faculty of Health Sciences, Ben-Gurion University of the Negev, Beer-Sheva, Israel; 4 Division of Cardiology, Soroka University Medical Center and The Faculty of Health Sciences, Ben-Gurion University of the Negev, Beer-Sheva, Israel; Shenzhen University, CHINA

## Abstract

**Background:**

Point-of-care ultrasonography (PoCUS) is a rapidly evolving discipline that aims to train non-cardiologists, non-radiologists clinicians in performing bedside ultrasound to guide clinical decision. Training of PoCUS is challenging, time-consuming and requires large amount of resources. The objective of our study was to evaluate if this training process can be simplified by allowing medical students self-train themselves with a web-based cardiac ultrasound software.

**Methods:**

A prospective, single blinded, cohort study, comparing performance of 29 medical students in performing a six-minutes cardiac ultrasound exam. Students were divided into two groups: self-learning group, using a combination of E-learning software and self-practice using pocket ultrasound device compared to formal, frontal cardiac ultrasound course.

**Results:**

All 29 students completed their designated courses and performed the six-minutes exam: 20 students participated in the frontal cardiac ultrasound course and 9 completed the self-learning course. The median (Q1,Q3) test score for the self-learning group was higher than the frontal course group score, 18 (15,19) versus 15 (12,19.5), respectively. Nevertheless, no statistically significant difference was found between the two study groups (p = 0.478). All students in the self-learning course group (9/9, 100%) and 16 (16/20, 80%) of students in the frontal ultrasound course group obtained correct alignment of the parasternal long axis view (p = 0.280).

**Conclusions:**

Self-learning students combining E-learning software with self-practice cardiac ultrasound were as good as students who received a validated, bedside, frontal cardiac ultrasound course. Our findings suggest that independent cardiac ultrasound learning, combining utilization of E–learning software and self-practice, is feasible. Self-E- learning of cardiac ultrasound may serve as an important, cost-effective adjunct to heavily resource consuming traditional teaching.

## Introduction

Physician skills in performing physical examination as well as medical educators’ ability to teach these skills are generally thought to be diminishing [1–3],[4–8].

We are entering new era in bedside physical examination and in medical education where cardiac ultrasound performed at the bedside by handheld and pocked ultrasound devices (PUDs) complement physical examination and improves bedside point of care diagnostic skills [9–14]. Point-of-care ultrasonography (PoCUS) is a new promising skill that is revolutionizing bedside physical examination [15]. To date several guidelines addressing PoCUS have been published from major organizations including the American Council on Education[12, 16], the American college of emergency physicians [17] and the European Association of Cardiovascular Imaging [18] With a wealth of data supporting the use of this technology and its effectiveness [5, 6, 15].

However, dedicated training in acquisition and interpretation of ultrasound images is needed to acquire and maintain competency, especially when the handheld echocardiographic examination is performed by non- echo cardiographers [19]. Teaching ultrasound is highly time-consuming and labor-intensive, traditionally necessitating bedside teaching in small groups. The ever- increasing need for proficient medical educators occurs in parallel with a trend for increasing medical school class sizes creates additional challenges. Medical school curricula are highly condensed, introducing new course, involving significant hands on training time may pose another problem. Moreover, hands-on courses may be very demanding to teaching faculty, many of whom are deeply involved in clinical practice and medical research. Medical schools face the challenge of creating novel teaching strategies for this new, important and advanced physical examination.

The objective of our study was to compare success rate in acquisition of basic cardiac ultrasound views, between medical students using computer based, self-learning cardiac ultrasound course, and students whom undertook formal, frontal structured cardiac ultrasound course.

## Methods

### Study population

Ethical approval for the data collection was obtained from the Ethics Committee of Soroka Medical Center. The committee approved a consent procedure in which participants expressed consent by taking a mandatory exam. All the students who participated were given assurance of confidentiality that the information gathered would be used exclusively for research purposes. We also indicated that participation in the study was anonymous and voluntary and filling in the written exam meant that the students were willing to participate in the study. All students could complete the course regardless of their consent to participate in the study. No sanctions from any kind were taken if a student refuses to participate. The frontal course and its’ 6-minute test were parts of the required syllabus regardless of the research. For the e- learning group this course was voluntary as well as the 6-minute exam. All students chose to participate in both. Prior to taking the 6 minute-exam all students were notified that this data might be used for research purposes.

**Fig 1:**
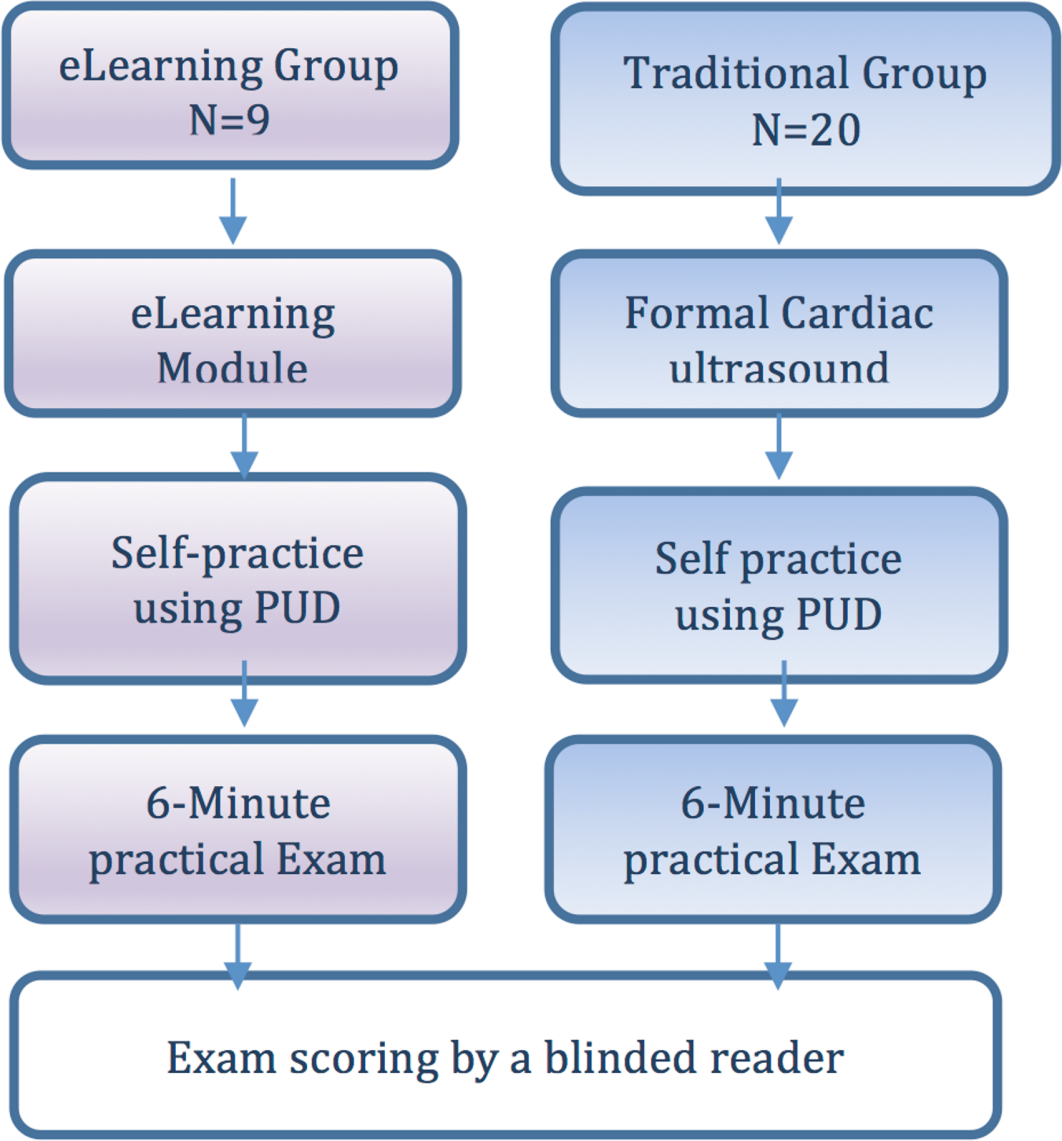
Schematic representation of the study design.

A total of 29 medical students were included in the study and were divided into two groups. The intervention group, titled as the E-learning cardiac ultrasound course group. In this group students were using online cardiac ultrasound course with no frontal teaching of any faculty member nor another teacher. The control group, titled as the frontal cardiac ultrasound course group, were students participated in a formal, structured cardiac ultrasound course that is part of 4^th^ year medical school clinical rotation curriculum (Fig 1).

### The E-learning cardiac ultrasound course

Group consisted of nine 3^rd^ year (out of six years medical school curriculum), volunteering medical students, given unlimited access to “eViews for Focused Cardiac Ultrasound”, one of four online learning courses offered by eMedical Academy (https://www.emedicalacademy.com). This web based course contains an e-views module teaching all formal echocardiographic views by integrating live videos, showing hand-probe-patient relationship, side to side with related real time echo views. Students had open access to the E-learning modules from the university as well as from their personal computers at home. Independent self-practice using a pocket ultrasound device (PUD) device was encouraged; for that purpose students received a brief instruction how to operate the PUD (Vscan from GE). Throughout the study period students in the E-learning group did not receive any kind of frontal, nor hands on teaching on how to perform cardiac ultrasound examination.

This group consisted of 3^rd^ year students while in the control group participated 4^th^ year students. This, because we compared 4^th^ year formal cardiac ultrasound course graduates (from the usual medical school curriculum) with students that were not exposed yet to cardiac ultrasound teaching from the 3^rd^ year.

### Computer based, self-learning course module

**Fig 2:**
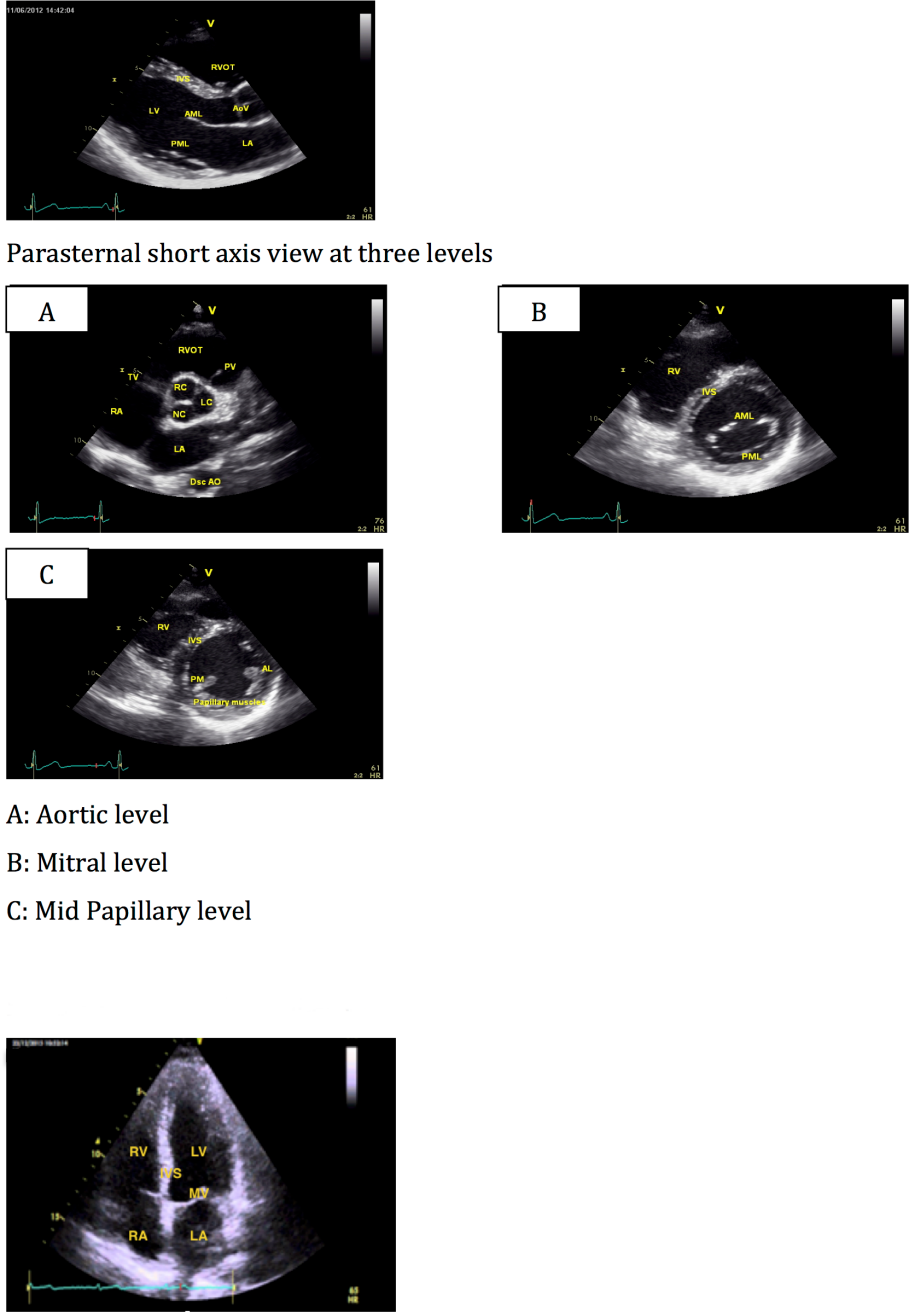
Trans thoracic echo views.

The module teaches transthoracic cardiac ultrasound views including parasternal long axis view (Fig 2A), parasternal short axis views (Fig 2A), apical 4-chamber view (Fig 2B), subcostal view (Fig 2C) including Inferior vena cava view (IVC) and illustrates proper probe orientation as well as sonographic anatomy, guidance on how to obtain each view and how to perform quality checks and trouble-shooting.

Apical 3-chamber and apical 2-chamber views were not included in the six-minute practical test.

### The frontal cardiac ultrasound course

This group consisted of twenty, randomly selected 4^th^-year medical students (in a six year program) from a class of 72 students. As this was a pilot study, designed to prove the concept of cardiac ultrasound self-learning, our convenience number for the control group was 20, allowing control to intervention group ratio of 2:1. The frontal course focused on obtaining the standard cardiac transthoracic ultrasound views and consisted of four hours of frontal lectures with an additional four hours of hands-on practice under supervision of cardiologists and sonographers[20]. Lectures focused on basic ultrasound physics, principals of two-dimensional ultrasound imaging, Doppler imaging, and the sonographic anatomy of the heart. The course was incorporated in an internal medicine rotation for students in their first year of clerkship. The students could then proceed to train with PUD autonomously. This course was shown to be feasible for training medical students and further described in a previous publication by our group [20]. All participating students gave informed consent for their participation in the study by agreeing to take the mandatory practical exam at the end of the course. The local Helsinki committee approved a consent procedure in which participants expressed consent by taking a mandatory exam.

### The six-minute exam

Following completion of the self-study period, students from both the self-learning group and frontal course group were evaluated using the same six-minute exam. The goal of this exam is to assess the students’ ability to perform a focused echocardiography examination and to obtain basic echocardiographic views [20].

Each student was given 6 minutes to obtain the main echocardiographic views in a predetermined order using a PUD: Parasternal long axis view (PLAX), Parasternal short axis view (PSAX) including aortic, mitral and mid-papillary level views, Apical 4- chamber view and subcostal view standard and focusing on the IVC. The exam ended after 6 minutes regardless of the quantity or quality of views acquired by the student.

The six-minute exam was performed under the supervision of a physician experienced in performing focused echocardiographic examination but each student decided when to acquire the required view and move to the next view.

Views obtained by students from both the self-learning and frontal course groups during the exam were captured as video files by cardiac ultrasound tutors, randomly scrambled and rated by two senior, echocardiography expert physicians blinded to the students group for evaluation. The raters were cardiologist and intensivist, each with daily cardiac ultrasound practical experience of over 5 years.

A checklist of key cardiac anatomical structures to be obtained in each echocardiographic view was employed to ensure a standardized assessment. For each cardiac view grade can be zero or one and maximal score possible is 28 (S1 Appendix). The views were obtained on healthy models who were pre-screened and had normal ultrasound windows.

PUD accessibility for self-practice was equal for the two groups. Time for self PUD practice was not predefined and was not measured in both groups.

### Statistical analysis

Success rates in the exam components are presented as frequency (%). Exam scores are reported as median (interquartile range [IQR]). We compared proportions using the Pearson χ^2^ test where applicable or Fisher’s Exact test. We compared exam scores using the Mann-Whitney U test. All *P* values are based on 2-tailed tests of significance. Intra-ratter scoring reliability was assessed by calculating absolute agreement using two-way mixed Interclass Correlation Coefficient (ICC), an index designed to express both degree of correlation and agreement between measurements. All computations were performed using SPSS version 24 (IBM Corp., Armonk, NY). Graphs were plotted using Microsoft Excel software for Windows.

## Results

All 29 students included in the study completed their training with either a frontal cardiac ultrasound course [20] or self-learning course [9] (Fig 1). All students completed the six-minute test, which was evaluated by two blinded, independent raters.

The median (Q1,Q3) test score for the self-learning group was higher than the frontal course group score [18 (15,19) vs 15 (12,19.5), S1 Appendix]. Nevertheless, no statistically significant difference was found between the two study groups (p = 0.508).

**Fig 3:**
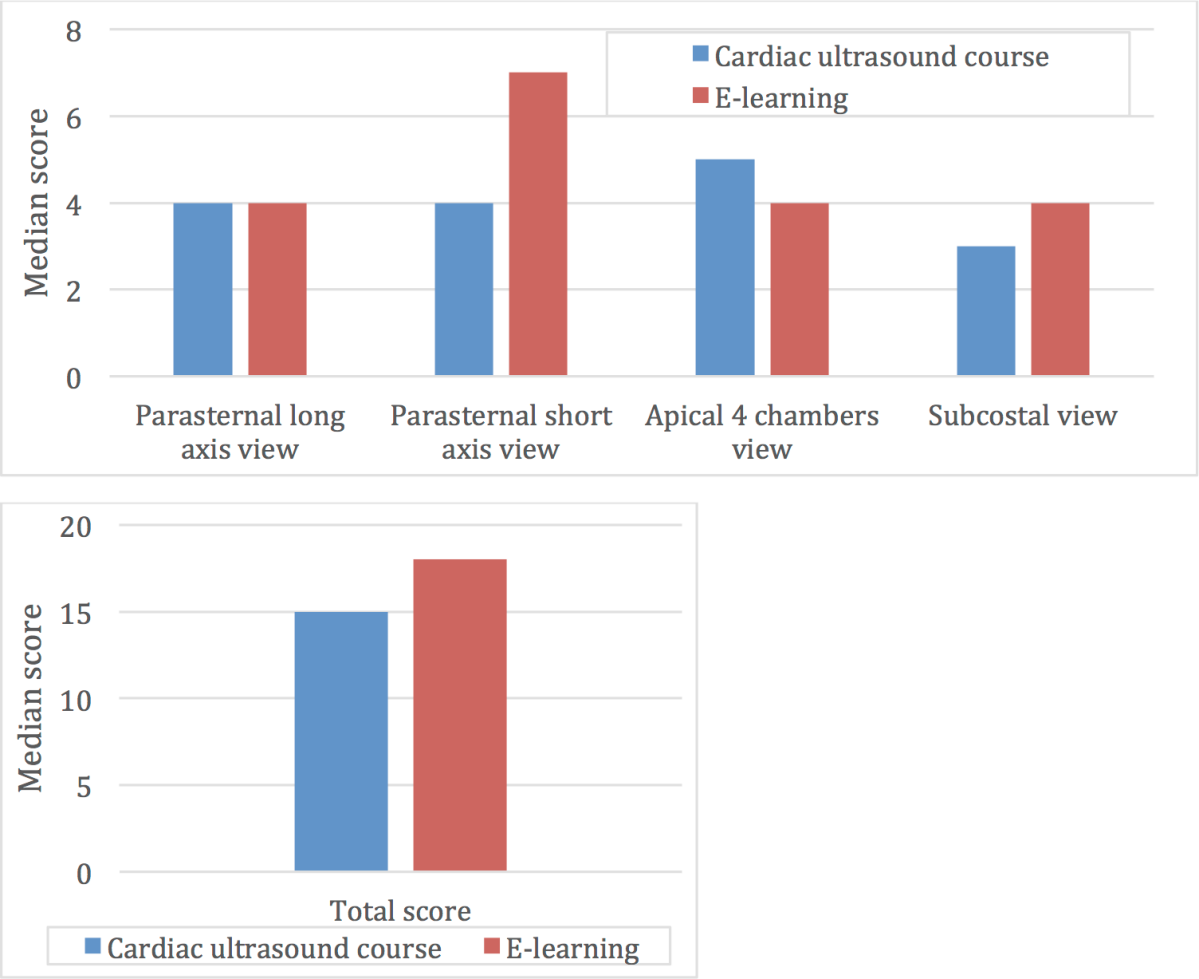
Comparison of median exam scores between eViews and Cardiac ultrasound course groups.

A comparison of the view alignment and visualization success rate for specific anatomical landmarks is shown in Table 1. Scores for the 6-minute test, assigned by independent raters are shown in Table 2 and Fig 3.

**Table 1:** Comparison of success rates between eViews and cardiac ultrasound course groups.

Variable		eViews n = 9	PoCUS course n = 20	P value
Parasternal long axis view	Correct alignment	9 (100%)	16 (80%)	0.280
	Endocardial demarcation	7 (77.8%)	18 (90%)	0.568
	Mitral valve visualization	8 (88.9%)	17 (85%)	1.0
	Aortic valve visualization	6 (66.7%)	14 (70%)	1.0
Parasternal short axis view	Aortic level demonstration	7 (77.8%)	11 (55%)	0.412
	Tricuspid valve visualization	4 (44.4%)	9 (45%)	1.0
	Pulmonic valve visualization	4 (44.4%)	9 (45%)	1.0
	Interatrial septum visualization	6 (66.7%)	8 (40%)	0.245
	Mitral level demonstration view	8 (88.9%)	12 (60%)	0.201
	Mitral valve visualization	8 (88.9%)	14 (70%)	0.382
	Mid-papillary level demonstration	9 (100%)	14 (70%)	0.137
	Papillary muscle visualization	8 (88.9%)	14 (70%)	0.382
Apical four chamber view	Left ventricle visualization	6 (66.7%)	16 (80%)	0.642
	Right ventricle visualization	7 (77.8%)	17 (85%)	0.633
	Mitral valve visualization	7 (77.8%)	18 (90%)	0.568
	Tricuspid valve visualization	4 (44.4%)	18 (90%)	**0.016**
	Right and left atria visualization	7 (77.8%)	16 (80%)	1.0
Apical two chamber view	Left ventricle visualization	8 (88.9%)	8 (40%)	**0.020**
	Mitral valve visualization	7 (77.8%)	11 (55%)	0.412
	Left atrium visualization	8 (88.9%)	11 (55%)	0.107
Apical 3 chamber view	Left ventricle visualization	4 (44.4%)	11 (55%)	0.700
	Mitral valve visualization	4 (44.4%)	12 (60%)	0.688
	Aortic valve visualization	4 (44.4%)	10 (50%)	1.0
Subcostal view	Right ventricle visualization	7 (77.8%)	13 (65%)	0.675
	Pericardial visualization	7 (77.8%)	17 (85%)	0.633
	Interatrial septum visualization	4 (44.4%)	9 (45%)	1.0
	Inferior vena cava visualization	7 (77.8%)	10 (50%)	0.234
	Inferior vena cava respiratory variation visualization	7 (77.8%)	9 (45%)	0.130

Data presented as median n (%). P values below significance threshold are highlighted.

**Table 2:** Comparison of exam scores between eViews and cardiac ultrasound course groups.

Group	E-learning group scores (n = 9)	Cardiac ultrasound course group scores (n = 20)	P value
Parasternal long axis view *(Max score 4)*	4 (3,4)	4 (3,4)	0.933
Parasternal short axis view *(Max score 8)*	7 (5,7)	4 (3,7)	0.142
Apical 4 chambers view *(Max score 5)*	4 (3,5)	5 (4.5,5)	0.061
Subcostal view *(Max score 5)*	4 (3,5)	3 (2,4)	0.226
**Total score** *(Max score 22)*	18 (15,19)	15 (12,19.5)	0.478

Data presented as median (Q1,Q3).

### Parasternal long axis view

All students in the self-learning course group (9/9, 100%) and 16 (16/20, 80%) of students in the frontal ultrasound course group obtained correct alignment of the PLAX view (p = 0.280). No statistically significant differences were found in visualization success rates for the endocardium, mitral and aortic valves in this view. Out of a maximal score 4, the median scores given to both groups were 4 (p = 0.933).

### Parasternal short axis view

Self-learning course students were more successful than the frontal course students at gaining the parasternal short although not achieving statistical significant difference (Table 2). Seven (78%) students from the self-learning course group and 11 (55%) students from the frontal ultrasound course group were successful in obtaining an aortic level PSAX view (p = 0.412). Eight (89%) and 12 (60%) could obtain a mitral level PSAX view from the self -learning and frontal ultrasound course groups respectively (p = 0.201). All self-learning students obtained a mid-papillary level PSAX view vs. 14 (70%) in the frontal ultrasound course group (p = 0.137), Table 1. The median score given for PSAX for the self-learning group was 7 vs. 4 for the frontal ultrasound course group (p = 0.142).

### Apical views

No statistically significant differences were found in students’ ability to demonstrate the right ventricle, mitral valve as well as both atria in A4Ch view. However, students from the frontal ultrasound-course were more successful in visualizing the tricuspid valve in A4ch compare to students from the self-learning group (90% vs. 44%, p = 0.016) Table 1.

### Subcostal views

Students were required to produce a traditional subcostal view as well as a tilted view of the inferior vena cava and attempt to demonstrate respiratory variation. (Table 1) More students in the self-learning course group were successful in demonstrating the inferior vena cava than the-frontal cardiac ultrasound course group but this difference did not achieve statistical significance (78% vs. 50%, p = 0.234).

The students in the self-learning course group were requested to report the time they have spent practicing using E–learning module. 7 out of 9 have reported total practice time, median practice time was 12.5 hours (6.67,17.0). Unfortunately, for both groups data on self PUD practice was not obtained.

### Inter-examiner correlation

Six-minute test scores were acquired from two independent examiners who were blinded to the student group assignment. The intra-class correlation coefficient (ICC) for the total exam score was 0.833 (p<0.0001) suggesting high correlation between the two raters.

## Discussion

This study shows that medical students were able to independently learn how to acquire cardiac ultrasound views by using an eLearning platform in combination with self-practice using a PUD. As well as we know, this is the first study designed to measure the efficacy of self-learning, cardiac ultrasound course taken by medical students. This, novice course, enabled medical students to succeed in the image acquisition of most of the basic ultrasound cardiac views, and perform as good as students who received a formal frontal course.

The E-learning cardiac ultrasound course performed better the six-minute cardiac ultrasound views test than the Frontal cardiac ultrasound course students, (better in total scores, PSAX view, the IVC and IVC respiratory variation views; Table 1, Table 2), but no statistical significant was found, possibly due to small sample size.

Ultrasound use is increasing in modern patient care. Portable ultrasound equipment is used by physicians as extension of the physical examination and allows for rapid bedside assessment and accurate management decisions based on ultrasound findings. While PoCUS has become the standard of care for many clinical scenarios, teaching PoCUS on a large scale poses a significant challenge for organizations with post-graduate physicians and clinicians, as well as for medical schools.

There is great deal of importance to the traditional bedside teaching, where the expert is passing his knowledge and experience to his students. Cardiac ultrasound especially demands hands on teaching, hands positioning and probe grip corrections as well as views interpretation. Small group teaching is essential at the bedside. For annual cycle of over 100 medical students, many teaching hours of highly cardiac ultrasound skilled physicians are needed. Hands-on courses are very demanding for the teaching faculty, many of whom are deeply involved in clinical practice and medical research. Medical schools face the challenge of creating novel teaching strategies for this new, important and advanced physical examination. Our data shows that self-learning of cardiac ultrasound is feasible and may complement and augment the traditional learning process, and by that, reduce the intensity of bedside teaching, saving human resources that are limited.

The ever-increasing body of knowledge required from medical students warrants independent learning, as medical school curriculum could not expand indefinitely. Many medical students expect professional tools for self-study in a modern medical education system. Recent studies demonstrated better outcomes for E–learning modules designed for medical students; E-learning can offer benefits regarding the reading of cerebral CT scans by students [21], other study found great improvement in dermatology knowledge when using E–learning techniques [22] and other showed improvement in bedside clinical evaluations and diagnostic skills [23, 24]. Our, self-learning course, shows the same trend: Self learning on students’ free time of cardiac ultrasound is feasible and can even be more effective than traditional frontal course (Tables 1 and 2).

This study has some strengths. This is a prospective study with blinding of the evaluating physicians. Also, we used an already validated course for novice medical students for the frontal ultrasound course group as well as validated 6 minutes exam [20]. Importantly, with all students being in their 3^rd^ and 4^th^ year of medical education, all were novice to ultrasound, so no bias exists, by having different starting ultrasound levels among the two compared groups. The students from the self- learning group had unlimited access of to the E-learning modules, facilitating independent learning, with no limitations. The students were encouraged to use the E-learning module but no arbitrary time commitment was required of them in order to perform the test. Both groups had free access to PUDs.

Our study also has several weaknesses. First, there may be selection bias, as the students from the self-learning group volunteered for the study and may have been motivated to study bedside cardiac ultrasound on their own, and therefore outperformed the fourth-year students who were evaluated with their class as part of a compulsory course, part of their medical school curriculum. Nevertheless, we wanted to show proof of concept, showing that medical students can teach themselves how to perform basic cardiac ultrasound study and we have. Second, students’ ultrasound image interpretation (identifying pathologies) was not assessed as it was not within the objectives of this study. Per design we planned it to be a pilot/proof of concept study, showing that medical students can teach themselves cardiac ultrasound when they have free access to a good e-learning platform and a hand held ultrasound device. We believe that with our relatively small sample size we were able to answer to the primary study question. Larger studies are needed. Small sample size may be potentially associated with insufficient statistical power to detect a difference between groups. Unfortunately, insufficient reports of self-study and independent hands-on practice times precluded our ability to measure any correlation between practice time and achievements in the 6-minute test.

## Conclusions

We found that students who trained on their own, with no bedside teaching, combining an E-learning module and self-cardiac ultrasound practice using PUD, were overall as good as students who received an already validated, bedside, frontal cardiac ultrasound course. Our findings suggest independent cardiac ultrasound learning is feasible and may serve as an important, cost-effective, modern adjunct to heavily resource consuming traditional cardiac ultrasound teaching.

## Supporting information

S1 Appendix 6-minute exam. (DOCX)
